# Case Report: Enfortumab Vedotin for Metastatic Urothelial Carcinoma: A Case Series on the Clinical and Histopathologic Spectrum of Adverse Cutaneous Reactions From Fatal Stevens-Johnson Syndrome/Toxic Epidermal Necrolysis to Dermal Hypersensitivity Reaction

**DOI:** 10.3389/fonc.2021.621591

**Published:** 2021-03-04

**Authors:** Paul V. Viscuse, Mario L. Marques-Piubelli, Meghan M. Heberton, Edwin Roger Parra, Amishi Y. Shah, Arlene Siefker-Radtke, Jianjun Gao, Sangeeta Goswami, Doina Ivan, Jonathan L. Curry, Matthew T. Campbell

**Affiliations:** ^1^ Division of Cancer Medicine-Hematology/Oncology Fellowship Program, The University of Texas MD Anderson Cancer Center, Houston, TX, United States; ^2^ Department of Translational Molecular Pathology, The University of Texas MD Anderson Cancer Center, Houston, TX, United States; ^3^ Department of Dermatology, The University of Texas MD Anderson Cancer Center, Houston, TX, United States; ^4^ Department of Genitourinary Medical Oncology, The University of Texas MD Anderson Cancer Center, Houston, TX, United States; ^5^ Department of Pathology, The University of Texas MD Anderson Cancer Center, Houston, TX, United States

**Keywords:** bladder cancer, enfortumab vedotin, SJS/TEN, urothelial cancer, adverse (side) effects, erythema multiform

## Abstract

Enfortumab vedotin is a Nectin-4 directed antibody-drug conjugate approved in metastatic urothelial carcinoma following progression on a platinum-containing chemotherapy and immune checkpoint blockade. On-target dermatologic toxicity may occur from Nectin-4 expression in the skin. We highlight a case of Stevens-Johnson Syndrome/Toxic Epidermal Necrolysis following enfortumab infusions that was ultimately fatal. The second case describes an erythema multiforme-like rash with interface dermatitis related to enfortumab. Dermatologic findings, immunohistochemistry studies, and immune profiling are detailed. These cases demonstrate the potentially catastrophic outcomes in some patients treated with enfortumab. Patients must be monitored for cutaneous toxicities with early involvement of dermatology and dermatopathology.

## Introduction

Enfortumab vedotin (EV) is an antibody-drug conjugate approved by the Food and Drug Administration in locally advanced or metastatic urothelial carcinoma following progression on platinum-containing chemotherapy and programmed death receptor-1 (PD-1) or programmed death-ligand 1 inhibitor (PD-L1). The antibody is directed at Nectin-4 and is linked to monomethyl auristatin E (MMAE), a potent antimitotic payload that blocks tubulin polymerization. The approval was based on a phase II study showing an overall response rate of 44% and median duration of response of 7.6 months (1). Preliminary results from the ongoing phase III EV-301 trial comparing EV to chemotherapy in previously treated patients with urothelial carcinoma have the primary end point of overall survival benefit (2). Dermatologic adverse events have been cited due to on-target toxicity from Nectin-4 expression in normal skin (3). Details of these events are not available, and the specific pathologic and immunologic findings have not been described.

We present our experience with enfortumab by describing a case of rapidly progressing Stevens-Johnson Syndrome/Toxic Epidermal Necrolysis (SJS/TEN) and a case of an erythema multiforme (EM)-like rash with histopathologic features of interface dermatitis related to EV.

## Case Description

### Patient 1

A 71-year-old male developed painless gross hematuria that persisted for a month. His past medical history was notable for compensated liver cirrhosis secondary to non-alcoholic fatty liver disease with portal hypertension, atrial fibrillation, and hypertension. Non-oncologic medications included aspirin, propranolol, and losartan. A contrast-enhanced CT of the abdomen and pelvis revealed a left kidney mass (4.6 × 5.9 × 4.0 cm) with a small (0.8 cm) left paracolic lymph node and several sub centimeter bibasilar solid pulmonary nodules.

**Figure 1:**
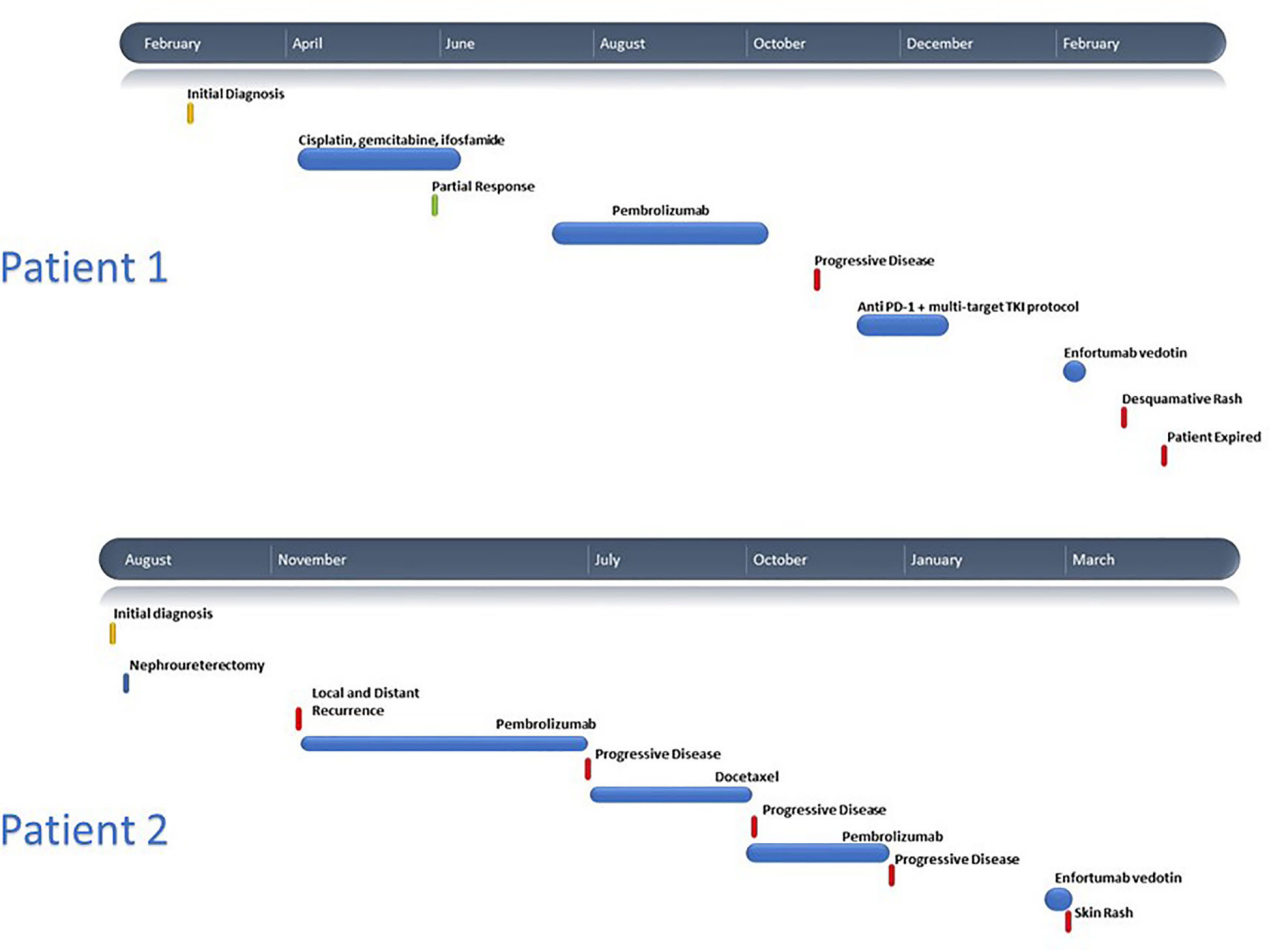
Clinical timelines for Patient 1 and Patient 2.

A CT-guided biopsy of the left kidney mass revealed high-grade urothelial carcinoma with invasion into the adjacent renal parenchyma. The lung nodules were suspicious for metastases, so he was started on cisplatin-based chemotherapy. He had a partial response after four cycles of treatment. He then received pembrolizumab IV 200 mg every 3 weeks and received a total of five cycles before discontinuation for disease progression. The patient enrolled on a clinical protocol with the combination of PD-1 antibody and an oral small molecule tyrosine kinase inhibitor targeting VEGFR2, KIT, TYRO3, AXL, and MER. Within one month, he developed grade IV pancreatitis which completely resolved with corticosteroid taper and cessation of therapy. He did not have any documented skin toxicity during this time. He initiated EV seven weeks from the last dose of immunotherapy and five weeks from the last dose of targeted therapy (**Figure 1**).

**Figure 2:**
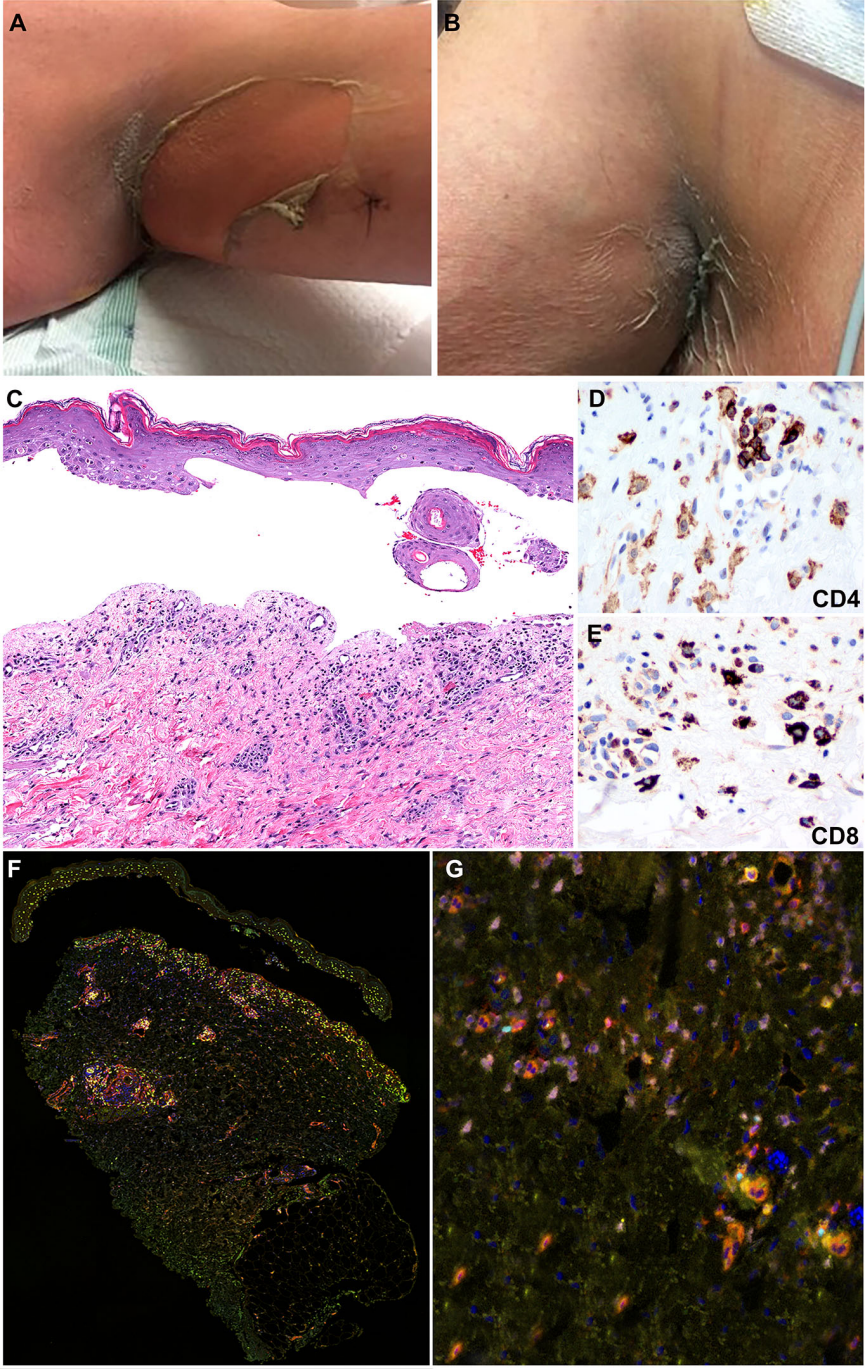
Clinicopathologic illustration of Patient 1. **(A, B)** Dusky erythematous patches and detachment of skin of the axillary vaults. **(C)** Patient 1 skin biopsy with epidermal detachment and blister cavity with scattered necrotic epidermal keratinocytes. An associated dermal superficial dermis shows interstitial and perivascular lymphohistiocytic infiltrate and few eosinophils and neutrophils (hematoxylin and eosin, original magnification, 40x). **(D, E)** Immunohistochemical studies show that the inflammatory infiltrates are composed of CD4+ T-cells with a subset of CD8+ T-cells (Immunohistochemistry, anti-CD4 and anti-CD8 x400). **(F, G)** Multiplex immunofluorescence (mIF) studies with immune-oncology toxicity panel of anti-CD3, anti-CD8, anti-FoxP3, anti-TBet, anti-Gata3, and anti-RORγT antibodies for patient 1. Stevens-Johnson syndrome/toxic epidermal necrolysis lesion shows inflammatory infiltrate in superficial dermis composed of CD3+ T-cells (orange) admixed with CD8+ T-cells (red). The infiltrate consists of increased numbers of Gata3+ cells (pink) and Th2 immunophenotype. Rare subsets of cells are positive for FoxP3 (cyan) and RORγT (green) corresponding to Tregs and Th17 immunophenotype, respectively.

He received EV 1.25 mg/kg on days 1 and 8 of the first cycle. Within 24 h of the day 8 infusion, he experienced diffuse pruritus. Four days after the infusion, he presented to the emergency department with fevers to 101°F and mouth pain but was otherwise hemodynamically stable with unremarkable cardiac and pulmonary exams. His dermatologic exam on admission revealed a small ulceration on the right lateral upper lip, well-demarcated erythema of the inferior tongue tip, and tender erythema of the axillae, flanks, inguinal region, and soles of feet (**Figures 2A, B**). He had flaccid ruptured bullae with approximately 11% of body surface area involvement that included the right heel, right posterior upper arm, and left forearm with positive Nikolsky sign. Eosinophil count and liver function tests were within normal limits. He was admitted and initiated on systemic steroids. Twelve days after treatment and 4 days after symptom onset, a punch biopsy of the left axillae confirmed subepidermal bulla with detached epidermis with scattered dyskeratotic cells and mixed dermal inflammatory infiltrate composed of lymphocytes, neutrophils, eosinophils, and macrophages (**Figure 2C**). The clinical and histologic findings were compatible with early changes of SJS/TEN.

**Figure 3:**
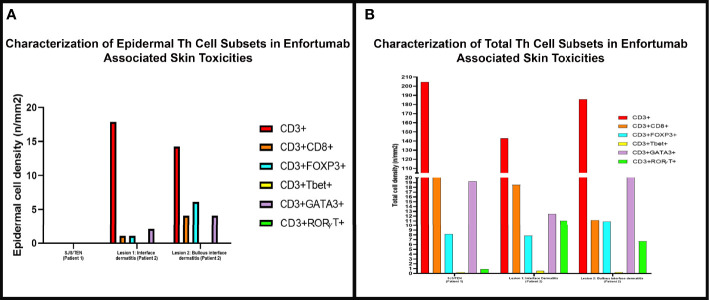
The density of skin infiltrating lymphocytes in the epidermis and skin biopsy by multiplex immunofluorescence (mIF) in patient 1 with Stevens-Johnson syndrome/toxic epidermal necrolysis SJS/TEN and patient 2 interface dermatitis toxicity (lesion 1) and bullous toxicity (lesion 2). **(A)** Skin infiltrating lymphocytes were absent in the epidermis in patient 1 with SJS/TEN. In patient 2, the interface dermatitis toxicity (lesion 1) and the bullous toxicity (lesion 2) exhibited CD3+ T-cells with variable density of CD8+ T-cells and Tregs and Th2 positive cells. **(B)** Examination of the total density of skin infiltrating lymphocytes by mIF analysis revealed that all three (SJS/TEN, interface dermatitis toxicity, and bullous toxicity) lesions analyzed consisted of CD3+ T-cells with a subset of CD8+ T-cells. All three lesions exhibited similar density of FoxP3+ cells and a minor population of TBet+ cells. The lesions from patient 2 exhibited higher density of RORγT+ cells compared to SJS/TEN lesion from patient 1.

The inflammatory infiltrate was composed of CD4+ and CD8+ T-cells admixed with MPO+ neutrophils and scattered CD68+ macrophages with absence of CD20+ B-cells (**Figures 2D, E**). Immune profiling with Vectra 3.0 spectral multiplex immunofluorescence (mIF) imaging system (PerkinElmer) and InForm 2.4.8 image analysis software were performed on the SJS/TEN skin biopsy utilizing a panel of antibodies to evaluate for the T-cell density (anti-CD3 and anti-CD4) and T helper (Th) cell immune response (Th1, anti-TBet; Th2, anti-Gata3; Th17, anti-ROR**γ**T; Tregs, anti-FoxP3). The density of skin infiltrating lymphocytes was composed predominantly of CD4+ T-cells with a subset of CD8+ T-cells (**Figures 2F, G**). There was absence of skin infiltrating lymphocytes in the epidermis. The Th immune profile consisted primarily of Th2 (Gata3+ cells) and Tregs (FoxP3+ cells) lymphocytes. A subset of CD4+ T-cells were Th17 (ROR**γ**T+ cells) and a minor population was Th1 (anti-TBet+ cells) (**Figure 3**).

ICU transfer with IV methylprednisolone treatment was initiated on day 3 for involvement of 18% total body surface area and high SCORETEN of 7. Antimicrobial therapy included cefepime, acyclovir, and mupirocin. He developed hypotension with a worsening rash, acute kidney injury, atrial fibrillation with rapid ventricular rate, hyponatremia, hyperglycemia, and anion-gap metabolic acidosis. Full thickness ulcers developed on the left upper arm, bilateral axillae, posterior ankles, right forearm, and scrotum with positive Nikolsky sign. He subsequently was transferred to the burn unit at a nearby hospital, but the patient expired several days later.

### Patient 2

**Figure 4:**
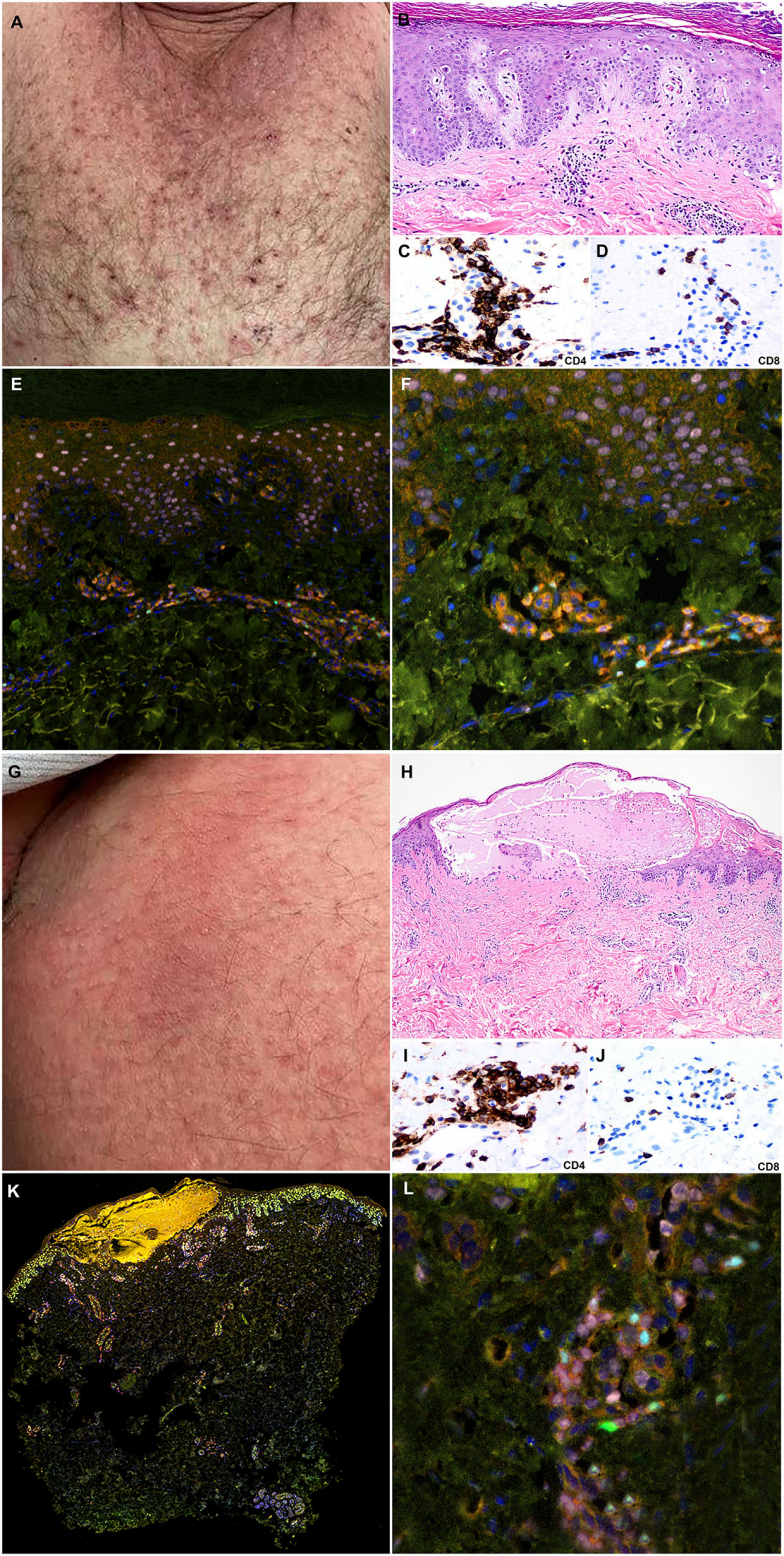
Clinicopathologic illustration of Patient 2. **(A)** Crusted thin red papules on the chest. **(B)** Patient 2 skin biopsy with interface dermatitis toxicity (lesion 1) with scattered dyskeratotic cells and the superficial perivascular dermal inflammatory infiltrate with eosinophils (hematoxylin and eosin, original magnification, 40x). **(C, D)** Immunohistochemical studies show that the inflammatory infiltrates are composed of CD4+ T-cells with a subset of CD8+ T-cells (Immunohistochemistry, anti-CD4 and anti-CD8, x400). **(E, F)** Multiplex immunofluorescence (mIF) studies with immune-oncology toxicity panel of anti-CD3, anti-CD8, anti-FoxP3, anti-TBet, anti-Gata3, and anti-RORγT antibodies. Patient 2, interface dermatitis toxicity (lesion 1) shows dermal inflammatory infiltrate that exhibits Th2 immunophenotype with Gata3+ cells (pink). There is a subset of Tregs, Foxp3+ cells (cyan), and Th17, RORγT positive cells (green) in the inflammatory infiltrate. **(G)** Erythematous patches on the abdomen. **(H)** Patient 2 skin biopsy with bullous toxicity (lesion 2) with scattered dyskeratotic cells (white arrows) and the superficial perivascular dermal inflammatory infiltrate with eosinophils and few neutrophils [hematoxylin and eosin (H&E), original magnification]. **(I, J)** Immunohistochemical studies show that the inflammatory infiltrates are composed of CD4+ T-cells with a subset of CD8+ T-cells (immunohistochemistry, anti-CD4 and anti-CD8, x400). **(K, L)** Multiplex immunofluorescence (mIF) studies with immune-oncology toxicity panel for patient 2; bullous toxicity (lesion 2) shows similar Th2 immunophenotype with Gata3+ cells (pink) and subsets of Tregs, Foxp3+ cells (cyan), and Th17, RORγT positive cells (green) [Vectra 3.0 spectral multiplex immunofluorescence (mIF) imaging system (PerkinElmer) and InForm 2.4.8 image analysis software Colors: Blue, DAPI; Red, CD3; Orange, CD8; Cyan, FOXP3; Yellow, TBet; Pink, GATA3; Green, RORγT].

A 77-year-old male presented with gross hematuria and flank pain in the setting of a left renal mass and underwent left nephroureterectomy with pathology confirming high grade papillary urothelial carcinoma. Two months later, he was found to have recurrence in the left nephrectomy bed, liver, lungs, lymph nodes, and bones with cisplatin ineligibility. He had recently progressed following treatment with docetaxel and, more recently, pembrolizumab which was discontinued approximately 2 months prior to his visit with no adverse effects (**Figure 1**). He was initiated on EV and subsequently developed a rash 2 days after his third infusion with tender erythema in the axillae, scrotum, and inguinal folds. Pruritic papules and vesicles of the chest and back, and bullae on the dorsal 2nd and 3rd digits of the left foot were observed (**Figure 4A**). Eighteen days after his first infusion, skin biopsy of chest (**Figure 4B**) and inguinal fold (**Figure 4H**) revealed bullous formation and interface dermatitis with dyskeratosis respectively. There were associated eosinophils and some neutrophils. The clinical and pathologic findings were compatible with EV-associated drug toxicity.

Immunohistochemical studies revealed perivascular dermal infiltrates with CD4+ and CD8+ T-cells (**Figures 4C, D, I, J**) and scattered CD68+ macrophages with few MPO+ neutrophils in the blister cavity and an absence of CD20+ B-cells. mIF studies revealed primarily CD4+ T-cell lymphocyte density with Tregs (FoxP3+ cells) in both lesions (**Figures 4E, F, K, L**). The interface dermatitis toxicity in the inguinal fold biopsy (lesion 1) exhibited a higher density of CD8+ lymphocytes compared to the bullous toxicity in the chest biopsy (lesion 2). There was also higher density Th17 (ROR**γ**T+ cells) in lesion 1 compared to lesion 2. The Th1 subset (TBet+ cells) of lymphocytes comprised of a small population of skin infiltrating lymphocytes with higher density of Th1 subset in the interface dermatitis toxicity (lesion 1) compared to bullous toxicity (lesion 2). In contrast, the density of Th2 lymphocytes (Gata3+ cells) was greater in lesion 2 compared to the lesion 1 (**Figure 3**).

Eosinophils were within normal limits. Alanine aminotransferase (ALT) and aspartate aminotransferase (AST) were elevated to 89 U/L and 99 U/L respectively. He had significant improvement in his rash and liver enzyme levels following 24–48 h of treatment with silver sulfadiazine cream, triamcinolone 0.1% ointment TID, and prednisone 60 mg daily. He continues EV without further complications.

## Discussion

Metastatic urothelial carcinoma has a poor prognosis with a median overall survival of less than 12 months. Approximately half of patients respond to first-line platinum-based chemotherapy and many patients are platinum-ineligible due to comorbidities (4, 5). Anti-PD-1/L1 therapy has been approved post-platinum with objective response rates up to 21% in the second-line setting and 24% in the first-line setting for platinum-ineligible patients (6, 7). Some patients who progress following chemotherapy and immunotherapy and demonstrate an FGFR alteration have the option to receive the FGFR 1-4 inhibitor erdafitinib (8). For the vast majority of patients, no standard therapeutic options remain outside of EV. EV is a fully human monoclonal antibody conjugated to a microtubule-disrupting agent monomethyl auristatin E (MMAE). It targets Nectin-4 which is a transmembrane protein involved in oncogenesis and is highly expressed in urothelial carcinoma. It received accelerated approval by the Food and Drug Administration in December 2019 based on results from the phase II EV201 study (1).

Of note, Nectin-4 is weakly to moderately expressed in skin with rashes occurring as an on-target toxicity. EV201 noted dermatologic adverse events in 48% of patients (25% grade 3 or higher) with onset typically seen in the first treatment cycle, consisting of weekly dosing on day 1, day 8, and day 15 out of 28 days. Most were described as “low grade”, “maculopapular”, and “diffuse”. Events were typically manageable with topical corticosteroids, oral antihistamines, systemic corticosteroids, and/or dose reductions/delays. However, one patient experienced a grade 3 rash reported as SJS 4 days after the initial dose with resolution after EV discontinuation and steroid treatment. There were no drug related deaths reported (1). Application of the Naranjo algorithm indicates probable causality and relationship of skin toxicity to EV in our cases (9). The proposed mechanism is targeting of Nectin-4 by EV with delivery of the MMAE payload to the skin resulting in the observed keratinocyte apoptosis. Alternatively, the dermatologic sequalae observed could be attributed solely to the MMAE payload without Nectin-4 direction. This is supported by the common occurrence of skin rash as an adverse event in trials evaluating other antibody-drug conjugates that incorporate MMAE, occurring in 31% of patients with classical Hodgkin lymphoma treated with brentuximab vedotin monotherapy, 44% of patients treated with glemutumumab vedotin, and 13–31% of patients treated with polatuzumab vedotin (10). Lastly, dermatologic toxicities from prior immune checkpoint inhibitor treatment cannot be entirely ruled out as delayed onset of SJS/TEN >8 weeks following exposure have been cited (11, 12). The histomorphologic features exhibit interface dermatitis with dyskeratosis (3). The extent of damage to the DEJ and subsequent blister formation and clinical manifestation of SJS/TEN appear variable between patients and among lesions biopsied in a patient (*e.g.*, patient 2). The composition of the immune infiltrate also appears variable among lesions though conclusions from this small sample size are limited. All lesions were composed of Th2 lymphocytes with a subset of Tregs and Th17 cells. All biopsies exhibited a subset of Th17 cells, which have been reported in peripheral blood and skin biopsies of patients with SJS/TEN, EM, and drug induced hypersensitivity reactions (13). *Ex vivo e*xpansion of skin infiltrating lymphocytes in patients with SJS/TEN, EM, and drug induced dermal hypersensitivity reaction (DHR) revealed the dynamic production of IL-17 with maximum concentration of IL-17 cells at 21 days after onset of skin reaction (13). Furthermore, increased Th17 cells in the peripheral blood and blister fluid of patients with SJS/TEN has been observed (14). Collectively, the composition of the immune infiltrate and Th17 cell subset may have a role in EV associated skin toxicities. The extent of skin infiltrating Th17 cells may be related to the timing of biopsy, onset of toxicity, and disease course.

SJS/TEN represent a spectrum of febrile mucocutaneous drug reactions (15). Categories are delineated based on BSA involvement: 1) SJS <10% BSA; 2) SJS/TEN overlap >10 to <30% BSA; 3) TEN >30% BSA (16). Rashes tend to present as dusky, red skin macules and/or patches with progression to widespread bullae, skin sloughing, and mucosal erosions with positive Nikolsky sign and associated fevers. A skin biopsy can help differentiate from other possible entities though it mainly represents a clinical diagnosis. Most cases are associated with medications, typically antibiotics, but also with allopurinol and anticonvulsants (17). More recently, RAF and immune checkpoint inhibition may be associated with SJS/TEN (18). Lesions typically occur 7-21 days after drug exposure but can occur within 2 days upon re-exposure. Management requires discontinuation of the offending agent. Supportive care with fluid resuscitation, electrolyte replacement, and nutrition should be provided in an intensive care setting. Use of non-adherent dressings provides topical skin care. There is otherwise limited evidence for other therapeutic interventions such as IVIG, IV cyclosporine, IV corticosteroids, and/or TNF-α antagonist such as etanercept (19–22). Survival often results in sequelae of scarring, pigmentation changes, and ocular complications. There is a high mortality rate of 0–9% in SJS, 3.9–19.4% in SJS/TEN, and 15-23% for TEN (23, 24). The SCORTEN score within 24 hours of admission and again on day 3 of hospitalization can aid prognostication. The score ranges from 0 to 7 with one point for each of the following: 1) Age 40 y or older; 2) pulse 120 bpm or more; 3) comorbid malignancy; 4) 10% or more body surface involvement; 5) serum urea >28 mg/dl; 6) serum glucose >252 mg/dl; 7) serum bicarbonate <20 mEq/L (25).

## Conclusion

With limited therapeutic options for patients with urothelial carcinoma following progression on platinum-based chemotherapy and immune checkpoint blockade, EV will see increased use. In patients with co-morbidities, low performance status, and laboratory values not acceptable for clinical trials, toxicity may be enhanced as compared to clinical trial participants. Since Nectin-4 is expressed in the skin, it is plausible that we will see increased skin toxicity from EV. Though it is not entirely clear if the catastrophic outcomes described in patient 1 are due to EV, vigilance is warranted in this subset of patients. These patients must be monitored to characterize the type of cutaneous toxicity with early involvement of dermatology and dermatopathology.

## Data Availability Statement

The raw data supporting the conclusions of this article will be made available by the authors, without undue reservation.

## Ethics Statement

Written informed consent was not obtained from the individual(s) for the publication of any potentially identifiable images or data included in this article.

## Author Contributions

PV and MM-P contributed equally to development of the manuscript. JC and MC contributed equally and provided expert oversight for the completion of the manuscript. MH provided dermatology input on manuscript and images. EP performed tissue studies for figures. AS, AS-R, JG, DI, and SG provided input on preparation of manuscript. All authors contributed to the article and approved the submitted version.

## Conflict of Interest

MC: Advisory Board/Honorarium: Seattle Genetics, Astellas, Eisai, AstraZeneca, Exelixis, EMD Serono, Pfizer Education grants: Roche, Bristol Myers Squibb, Pfizer Research grants: AstraZeneca, Exelixis, Janssen, Pfizer, EMD Serono.

The remaining authors declare that the research was conducted in the absence of any commercial or financial relationships that could be construed as a potential conflict of interest.
